# Supplementary material for: A Likelihood Approach for Real-Time Calibration of Stochastic Compartmental Epidemic Models
Source: PLoS Comput Biol. 2017 Jan 17;13(1):e1005257. doi: 10.1371/journal.pcbi.1005257 (PMC5240920; doi:10.1371/journal.pcbi.1005257)
Supplement: S1 File — (TAR.GZ) [file pcbi.1005257.s014.tar.gz › HSPH_Online-SI-Revision/Additions/MSS11/n100-extreme-SEITR/n100-extreme-SEITR_NEW2_table-8.pdf]

1	newly 3 weeks spec	2187.	1764.59	{1381., 2290.}	29.6658	0.144052	newly 3 weeks cumul	3760.	3180.73	{2583., 4001.}	25.5476	0.12025	newly 5 weeks spec	7106.	5052.16	{4147., 7097.}	33.7079	0.177851	newly 5 weeks cumul	14 965.	11 366.3	{9175., 15 275.}	31.4839	0.159279	newly 59 weeks cumul	91 886.	83 903.5	{56 339., 88 809.}	8.72573	0.0441963
2	newly 3 weeks spec	265.	27.56	{1., 39.}	95.8566	1.61226	newly 3 weeks cumul	343.	43.75	{4., 73.}	93.3907	1.29773	newly 5 weeks spec	3034.	196.57	{0., 126.}	99.3827	2.40873	newly 5 weeks cumul	4334.	316.62	{5., 262.}	98.7536	1.94761	newly 66 weeks cumul	99 017.	64 129.9	{5., 91 842.}	35.2335	0.85315
3	newly 3 weeks spec	257.	1251.45	{1., 769.}	497.638	0.96951	newly 3 weeks cumul	379.	1445.94	{9., 916.}	391.478	0.784241	newly 5 weeks spec	2684.	3790.66	{7., 11 346.}	162.858	1.22906	newly 5 weeks cumul	3894.	8146.79	{16., 20 695.}	228.503	1.04808	newly 68 weeks cumul	98 993.	69 674.5	{60., 92 596.}	29.6168	0.575933
4	newly 3 weeks spec	698.	429.05	{30., 887.}	69.2507	0.663777	newly 3 weeks cumul	1030.	703.61	{93., 1384.}	62.7117	0.491638	newly 5 weeks spec	3933.	1941.31	{45., 4119.}	76.8703	0.94233	newly 5 weeks cumul	6733.	3631.56	{162., 7597.}	74.4438	0.729286	newly 66 weeks cumul	96 862.	44 170.2	{13 788., 61 256.}	54.3988	0.571635
5	newly 3 weeks spec	187.	499.55	{0., 58.}	347.439	1.81552	newly 3 weeks cumul	237.	695.67	{2., 86.}	369.582	1.4319	newly 5 weeks spec	3231.	459.1	{0., 422.}	102.394	2.67158	newly 5 weeks cumul	4188.	1421.84	{2., 735.}	120.107	2.18145	newly 68 weeks cumul	98 880.	48 997.	{3., 91 786.}	50.4481	1.45419
6	newly 3 weeks spec	323.	94.97	{1., 97.}	103.39	1.40203	newly 3 weeks cumul	460.	128.54	{8., 161.}	98.9522	1.16771	newly 5 weeks spec	3690.	603.79	{0., 834.}	100.549	2.00343	newly 5 weeks cumul	5281.	1037.94	{11., 1263.}	101.915	1.67537	newly 67 weeks cumul	95 041.	69 479.2	{23., 93 469.}	26.8955	0.582659
7	newly 3 weeks spec	2336.	2060.8	{1722., 2502.}	20.1926	0.095927	newly 3 weeks cumul	4235.	3749.91	{3203., 4571.}	18.7568	0.0891977	newly 5 weeks spec	6848.	5576.99	{4828., 7007.}	23.1824	0.117403	newly 5 weeks cumul	15 415.	12 886.7	{11 114., 15 975.}	22.221	0.110059	newly 59 weeks cumul	97 367.	79 986.2	{54 700., 85 758.}	17.8508	0.0939911
8	newly 3 weeks spec	476.	302.75	{27., 345.}	98.7164	0.68579	newly 3 weeks cumul	697.	424.45	{63., 521.}	90.0961	0.612722	newly 5 weeks spec	4729.	1774.32	{81., 2452.}	95.5174	1.00801	newly 5 weeks cumul	7052.	3148.16	{205., 3937.}	98.1756	0.909206	newly 66 weeks cumul	99 432.	81 674.4	{60 378., 93 259.}	17.8591	0.0993489
9	newly 3 weeks spec	88.	14.	{0., 23.}	92.2955	1.54312	newly 3 weeks cumul	112.	21.09	{1., 42.}	88.8661	1.18792	newly 5 weeks spec	2227.	130.3	{0., 121.}	97.7611	2.59172	newly 5 weeks cumul	2748.	192.78	{2., 203.}	97.147	2.0802	newly 68 weeks cumul	99 354.	55 207.1	{2., 91 188.}	44.434	1.36741
10	newly 3 weeks spec	982.	1058.47	{683., 1511.}	24.3493	0.0995319	newly 3 weeks cumul	1566.	1659.64	{1107., 2278.}	22.6973	0.0938873	newly 5 weeks spec	4646.	5204.99	{3281., 7051.}	26.9451	0.107712	newly 5 weeks cumul	8317.	9349.93	{6027., 12 847.}	26.6792	0.105773	newly 64 weeks cumul	99 174.	87 166.8	{80 706., 91 762.}	12.1072	0.0568212
11	newly 3 weeks spec	578.	212.86	{89., 324.}	63.6263	0.491546	newly 3 weeks cumul	848.	376.95	{171., 574.}	56.5248	0.400532	newly 5 weeks spec	4035.	848.05	{316., 1374.}	78.9827	0.755216	newly 5 weeks cumul	6425.	1651.49	{694., 2573.}	74.2959	0.655223	newly 63 weeks cumul	99 600.	84 332.8	{71 289., 92 247.}	15.3285	0.0769425
12	newly 3 weeks spec	936.	404.01	{196., 642.}	57.9562	0.433599	newly 3 weeks cumul	1372.	647.31	{352., 1015.}	53.6859	0.380161	newly 5 weeks spec	5911.	2069.83	{785., 3656.}	67.0233	0.564518	newly 5 weeks cumul	9781.	3655.74	{1563., 6179.}	64.4159	0.517709	newly 64 weeks cumul	98 924.	85 175.4	{77 965., 93 576.}	13.8982	0.0731036
13	newly 3 weeks spec	632.	358.46	{142., 575.}	52.5601	0.384712	newly 3 weeks cumul	864.	552.27	{243., 871.}	46.0544	0.300777	newly 5 weeks spec	4789.	2178.94	{551., 3815.}	63.7281	0.531655	newly 5 weeks cumul	7468.	3641.69	{1094., 6235.}	60.757	0.465877	newly 65 weeks cumul	99 507.	83 722.2	{71 124., 92 853.}	15.863	0.118103
14	newly 3 weeks spec	1910.	1664.09	{1472., 1819.}	13.4267	0.0638196	newly 3 weeks cumul	3449.	3157.52	{2839., 3420.}	9.50072	0.0441011	newly 5 weeks spec	5421.	4307.86	{3885., 4670.}	20.5866	0.101257	newly 5 weeks cumul	12 064.	10 229.5	{9263., 11 092.}	15.5279	0.0742344	newly 58 weeks cumul	96 176.	82 107.2	{81 608., 82 220.}	14.6282	0.0687384
15	newly 3 weeks spec	1491.	1849.51	{908., 2531.}	40.2609	0.16182	newly 3 weeks cumul	2425.	3003.39	{1623., 4060.}	39.2944	0.155749	newly 5 weeks spec	6031.	7037.78	{3870., 9455.}	34.2238	0.152374	newly 5 weeks cumul	11 560.	13 911.9	{6892., 18 733.}	36.6677	0.154308	newly 59 weeks cumul	97 278.	65 729.6	{54 170., 71 141.}	32.4312	0.175861
16	newly 3 weeks spec	1486.	1472.34	{1038., 1879.}	25.4751	0.107119	newly 3 weeks cumul	2628.	2556.54	{1776., 3217.}	23.4117	0.101835	newly 5 weeks spec	4920.	4543.26	{3264., 6398.}	22.8711	0.112159	newly 5 weeks cumul	10 361.	9828.62	{6893., 13 416.}	22.9557	0.10675	newly 62 weeks cumul	92 728.	74 688.5	{36 701., 93 598.}	20.1985	0.124672
17	newly 3 weeks spec	187.	36.37	{0., 27.}	108.187	1.8709	newly 3 weeks cumul	247.	47.6	{2., 42.}	105.336	1.52427	newly 5 weeks spec	2188.	319.44	{0., 144.}	107.977	2.65075	newly 5 weeks cumul	3077.	520.11	{2., 231.}	109.266	2.14707	newly 68 weeks cumul	93 253.	56 820.7	{3., 91 853.}	39.1302	1.13253
18	newly 3 weeks spec	162.	25.09	{1., 49.}	87.8333	1.34354	newly 3 weeks cumul	222.	40.86	{4., 89.}	84.6847	1.06491	newly 5 weeks spec	2807.	166.93	{0., 243.}	94.0531	2.17772	newly 5 weeks cumul	3654.	270.38	{7., 430.}	92.7893	1.76295	newly 67 weeks cumul	99 640.	65 890.1	{10., 93 504.}	33.8718	0.690648
19	newly 3 weeks spec	726.	265.52	{136., 331.}	66.0716	0.500154	newly 3 weeks cumul	1060.	469.18	{246., 590.}	59.1132	0.409002	newly 5 weeks spec	4054.	1038.44	{454., 1324.}	76.1046	0.676477	newly 5 weeks cumul	6766.	2041.63	{984., 2538.}	71.9771	0.592822	newly 62 weeks cumul	99 318.	82 359.3	{60 724., 92 619.}	17.0751	0.0876593
20	newly 3 weeks spec	300.	586.04	{7., 870.}	205.687	0.797689	newly 3 weeks cumul	402.	698.93	{28., 1099.}	178.803	0.648361	newly 5 weeks spec	3692.	3500.88	{28., 11 019.}	119.134	1.02169	newly 5 weeks cumul	5182.	6179.44	{56., 15 591.}	140.662	0.902641	newly 67 weeks cumul	98 887.	81 956.9	{67 523., 93 631.}	17.1207	0.260859
21	newly 3 weeks spec	120.	30.31	{0., 87.}	81.7417	1.23894	newly 3 weeks cumul	157.	47.43	{3., 126.}	79.2548	0.954213	newly 5 weeks spec	2417.	243.79	{0., 831.}	90.2089	2.07527	newly 5 weeks cumul	3205.	373.39	{3., 1286.}	88.8796	1.67947	newly 68 weeks cumul	99 034.	62 599.1	{3., 92 522.}	36.7903	0.921008
22	newly 3 weeks spec	1137.	2112.6	{1340., 2828.}	87.3386	0.252178	newly 3 weeks cumul	1829.	3180.76	{2068., 4176.}	75.825	0.228902	newly 5 weeks spec	5958.	9750.49	{6444., 12 599.}	64.9475	0.203732	newly 5 weeks cumul	10 630.	18 003.2	{11 852., 23 576.}	70.8374	0.216741	newly 64 weeks cumul	96 878.	87 977.9	{79 307., 93 884.}	9.18691	0.0429305
23	newly 3 weeks spec	1235.	3747.48	{792., 8246.}	220.269	0.431545	newly 3 weeks cumul	1958.	5355.82	{1259., 11 747.}	190.332	0.3913	newly 5 weeks spec	6598.	12 377.8	{3879., 20 029.}	108.986	0.339491	newly 5 weeks cumul	11 490.	25 875.4	{7411., 42 923.}	144.387	0.387082	newly 65 weeks cumul	88 617.	70 412.1	{50 566., 82 997.}	20.9094	0.110555
24	newly 3 weeks spec	102.	8.45	{0., 14.}	94.2647	1.79572	newly 3 weeks cumul	138.	14.98	{2., 29.}	91.7391	1.33255	newly 5 weeks spec	2027.	30.95	{0., 52.}	98.4731	2.86432	newly 5 weeks cumul	2665.	61.64	{2., 116.}	97.6871	2.23604	newly 67 weeks cumul	98 866.	45 103.6	{2., 89 103.}	54.3791	1.76812
25	newly 3 weeks spec	917.	796.54	{540., 1019.}	21.0971	0.10289	newly 3 weeks cumul	1410.	1295.03	{922., 1642.}	18.1255	0.0850001	newly 5 weeks spec	3941.	3591.07	{2549., 4496.}	18.5336	0.0886534	newly 5 weeks cumul	7263.	6640.37	{4650., 8489.}	18.612	0.088189	newly 63 weeks cumul	94 839.	88 241.8	{83 502., 93 608.}	6.9562	0.0316933
26	newly 3 weeks spec	2138.	2879.97	{2549., 3153.}	35.5748	0.130977	newly 3 weeks cumul	3825.	5007.62	{4443., 5513.}	31.8886	0.119227	newly 5 weeks spec	6408.	8192.48	{7563., 8855.}	29.2166	0.111069	newly 5 weeks cumul	14 163.	18 398.7	{16 454., 19 856.}	31.1178	0.117094	newly 61 weeks cumul	87 857.	88 365.2	{82 538., 93 156.}	7.16156	0.0331119
27	newly 3 weeks spec	1999.	1797.17	{1560., 2129.}	12.5018	0.0593348	newly 3 weeks cumul	3536.	3138.99	{2759., 3639.}	12.6094	0.0599224	newly 5 weeks spec	6511.	5759.07	{5004., 6632.}	13.3926	0.0642067	newly 5 weeks cumul	13 890.	12 268.5	{10 645., 14 170.}	13.2896	0.0634726	newly 61 weeks cumul	98 347.	88 820.2	{82 961., 93 007.}	9.68697	0.0459236
28	newly 3 weeks spec	310.	20 534.6	{878., 43 939.}	6529.66	1.61842	newly 3 weeks cumul	474.	25 672.1	{1218., 56 673.}	5320.81	1.48651	newly 5 weeks spec	2971.	14 479.8	{2724., 27 448.}	400.334	0.740224	newly 5 weeks cumul	4384.	64 352.9	{14 088., 89 810.}	1375.75	1.15162	newly 68 weeks cumul	89 828.	77 453.	{49 755., 93 850.}	16.5089	0.159365
29	newly 3 weeks spec	609.	811.75	{342., 1192.}	53.7291	0.183431	newly 3 weeks cumul	951.	1215.91	{546., 1778.}	47.4648	0.166412	newly 5 weeks spec	3642.	4678.51	{2018., 7360.}	53.8652	0.202985	newly 5 weeks cumul	6162.	7973.88	{3418., 12 330.}	53.4288	0.19386	newly 65 weeks cumul	99 052.	82 004.6	{70 221., 92 199.}	17.2105	0.0915905
30	newly 3 weeks spec	109.	70.67	{0., 25.}	143.367	1.75229	newly 3 weeks cumul	138.	83.08	{1., 40.}	133.304	1.29451	newly 5 weeks spec	1649.	500.41	{0., 122.}	118.042	2.55468	newly 5 weeks cumul	2255.	923.71	{2., 221.}	127.205	2.04726	newly 68 weeks cumul	99 316.	55 142.4	{2., 91 964.}	44.4779	1.34326
31	newly 3 weeks spec	3137.	2606.71	{1929., 3278.}	19.3201	0.100085	newly 3 weeks cumul	6051.	5550.4	{4183., 6600.}	14.6673	0.0723142	newly 5 weeks spec	6713.	4233.29	{3481., 4838.}	36.9389	0.205659	newly 5 weeks cumul	17 548.	13 293.3	{10 344., 15 862.}	24.3881	0.128113	newly 58 weeks cumul	87 424.	36 197.2	{32 354., 39 158.}	58.5958	0.38733
32	newly 3 weeks spec	1418.	1232.47	{792., 1712.}	26.6199	0.142111	newly 3 weeks cumul	2225.	1983.86	{1313., 2689.}	24.489	0.127469	newly 5 weeks spec	5244.	5362.96	{3414., 7600.}	25.8192	0.123453	newly 5 weeks cumul	10 289.	10 063.7	{6479., 14 079.}	25.0775	0.123538	newly 64 weeks cumul	98 867.	81 214.3	{56 863., 92 032.}	17.855	0.0933816
33	newly 3 weeks spec	501.	7499.67	{3., 30 739.}	1459.87	1.1895	newly 3 weeks cumul	673.	9575.88	{20., 33 694.}	1381.52	0.955219	newly 5 weeks spec	5187.	11 246.6	{0., 27 880.}	190.98	1.21602	newly 5 weeks cumul	7427.	32 521.6	{24., 81 601.}	406.055	1.06583	newly 68 weeks cumul	99 003.	62 305.2	{25., 93 026.}	37.0673	0.705471
34	newly 3 weeks spec	1440.	1675.83	{976., 2456.}	34.2535	0.140707	newly 3 weeks cumul	2363.	2541.92	{1535., 3822.}	28.6686	0.125104	newly 5 weeks spec	6918.	8225.86	{4969., 11 475.}	35.558	0.149424	newly 5 weeks cumul	12 627.	14 866.6	{8913., 20 705.}	34.6199	0.143787	newly 64 weeks cumul	95 674.	82 375.1	{59 294., 92 113.}	13.9002	0.0743534
35	newly 3 weeks spec	179.	551.41	{0., 143.}	368.866	1.55354	newly 3 weeks cumul	229.	611.39	{2., 220.}	321.568	1.16733	newly 5 weeks spec	2514.	1257.29	{0., 1418.}	122.275	2.20671	newly 5 weeks cumul	3425.	3064.67	{3., 2173.}	159.674	1.77422	newly 68 weeks cumul	95 744.	59 505.6	{3., 93 290.}	37.8493	1.10521
36	newly 3 weeks spec	451.	290.9	{144., 381.}	45.0244	0.269621	newly 3 weeks cumul	672.	471.37	{239., 606.}	39.686	0.223803	newly 5 weeks spec	3163.	1490.27	{585., 2075.}	60.6775	0.432089	newly 5 weeks cumul	5077.	2634.25	{1156., 3558.}	56.3937	0.377177	newly 64 weeks cumul	99 501.	87 048.8	{82 803., 93 820.}	12.5147	0.0637948
37	newly 3 weeks spec	142.	2.7	{0., 6.}	98.0986	2.21612	newly 3 weeks cumul	189.	6.18	{1., 13.}	96.7302	1.72654	newly 5 weeks spec	2178.	5.8	{0., 14.}	99.7337	3.29707	newly 5 weeks cumul	2952.	15.97	{2., 40.}	99.459	2.61842	newly 65 weeks cumul	99 424.	16 195.9	{2., 62 878.}	83.7103	2.66531
38	newly 3 weeks spec	1570.	1742.51	{1360., 2123.}	17.765	0.0708361	newly 3 weeks cumul	2591.	2884.78	{2322., 3506.}	16.7102	0.0664538	newly 5 weeks spec	6266.	6625.52	{5009., 7774.}	15.0587	0.0633769	newly 5 weeks cumul	12 039.	13 112.5	{10 070., 15 714.}	16.5245	0.0674349	newly 62 weeks cumul	93 543.	89 217.5	{86 363., 93 294.}	4.62542	0.0207205
39	newly 3 weeks spec	955.	1085.2	{650., 1627.}	34.756	0.136559	newly 3 weeks cumul	1540.	1657.15	{1056., 2424.}	30.9435	0.12752	newly 5 weeks spec	4980.	5779.85	{3622., 9316.}	35.1139	0.135212	newly 5 weeks cumul	8707.	10 117.8	{6320., 15 821.}	35.3114	0.135357	newly 64 weeks cumul	97 320.	85 644.5	{71 344., 92 852.}	11.9971	0.0589621
40	newly 3 weeks spec	1152.	8433.1	{2163., 16 518.}	633.543	0.747504	newly 3 weeks cumul	1821.	11 250.8	{3209., 21 473.}	519.577	0.674336	newly 5 weeks spec	6088.	18 863.7	{11 199., 25 028.}	210.954	0.471273	newly 5 weeks cumul	10 814.	46 580.	{24 840., 68 345.}	331.378	0.605268	newly 65 weeks cumul	99 384.	78 966.3	{50 691., 93 111.}	20.5442	0.108555
41	newly 3 weeks spec	84.	47.11	{0., 81.}	105.06	1.25455	newly 3 weeks cumul	118.	68.3	{2., 108.}	101.542	0.954301	newly 5 weeks spec	1333.	466.17	{0., 1046.}	105.24	2.03656	newly 5 weeks cumul	1781.	685.46	{2., 1494.}	105.37	1.5856	newly 68 weeks cumul	96 330.	55 486.3	{2., 92 179.}	42.3997	1.3343
42	newly 3 weeks spec	2385.	2651.75	{2311., 3046.}	15.2918	0.0632751	newly 3 weeks cumul	4744.	4993.28	{4338., 5660.}	10.7028	0.0461998	newly 5 weeks spec	6151.	6445.3	{5701., 7225.}	11.961	0.0537492	newly 5 weeks cumul	14 873.	15 776.2	{13 738., 17 828.}	12.0821	0.0524503	newly 59 weeks cumul	96 441.	77 120.5	{67 252., 89 932.}	20.0335	0.105137
43	newly 3 weeks spec	942.	1787.96	{1182., 2446.}	90.327	0.262061	newly 3 weeks cumul	1522.	2704.94	{1848., 3591.}	78.2076	0.236018	newly 5 weeks spec	4281.	8534.65	{5939., 11 380.}	99.5307	0.28469	newly 5 weeks cumul	7799.	15 516.7	{10 620., 20 804.}	99.1694	0.282749	newly 64 weeks cumul	98 631.	90 823.2	{85 066., 93 891.}	7.91621	0.0363465
44	newly 3 weeks spec	717.	351.64	{199., 512.}	51.06	0.342906	newly 3 weeks cumul	1097.	573.66	{325., 837.}	47.8086	0.309668	newly 5 weeks spec	5449.	1759.69	{848., 2840.}	67.8405	0.538296	newly 5 weeks cumul	8791.	3132.84	{1600., 4914.}	64.4004	0.488369	newly 64 weeks cumul	99 299.	86 501.4	{77 798., 93 441.}	12.8879	0.0614871
45	newly 3 weeks spec	514.	1201.82	{165., 1698.}	166.016	0.360556	newly 3 weeks cumul	734.	1567.39	{240., 2250.}	144.969	0.321246	newly 5 weeks spec	4552.	7273.67	{1357., 15 367.}	93.3403	0.359275	newly 5 weeks cumul	6924.	12 172.3	{2192., 24 589.}	109.102	0.34939	newly 66 weeks cumul	99 357.	86 573.1	{76 639., 93 691.}	12.8666	0.0998666
46	newly 3 weeks spec	247.	8.46	{0., 17.}	96.5749	2.02731	newly 3 weeks cumul	335.	14.91	{1., 27.}	95.5493	1.68814	newly 5 weeks spec	2717.	73.35	{0., 79.}	97.346	2.74343	newly 5 weeks cumul	3855.	110.	{2., 180.}	97.1466	2.29659	newly 68 weeks cumul	99 095.	54 894.3	{3., 91 202.}	44.6044	1.22857
47	newly 3 weeks spec	977.	457.33	{287., 604.}	53.1904	0.348803	newly 3 weeks cumul	1597.	861.5	{563., 1125.}	46.0551	0.282804	newly 5 weeks spec	4864.	1415.59	{754., 2013.}	70.8966	0.565781	newly 5 weeks cumul	8755.	3091.23	{1808., 4245.}	64.6918	0.475535	newly 63 weeks cumul	96 045.	85 566.9	{79 456., 92 197.}	10.9096	0.0508235
48	newly 3 weeks spec	364.	506.79	{151., 937.}	70.8654	0.254317	newly 3 weeks cumul	527.	721.63	{233., 1294.}	66.2334	0.237753	newly 5 weeks spec	3183.	4034.75	{1019., 7870.}	70.3069	0.289337	newly 5 weeks cumul	4879.	6276.53	{1649., 11 676.}	69.3357	0.276742	newly 66 weeks cumul	97 052.	85 904.7	{71 164., 93 461.}	11.486	0.0555262
49	newly 3 weeks spec	1900.	2148.93	{1250., 3174.}	33.8405	0.138333	newly 3 weeks cumul	3162.	3498.92	{2025., 5086.}	31.1417	0.129623	newly 5 weeks spec	7611.	7619.13	{4177., 10 714.}	26.4195	0.123689	newly 5 weeks cumul	14 764.	15 564.	{8611., 22 287.}	29.0021	0.127744	newly 64 weeks cumul	95 259.	63 340.7	{54 309., 71 271.}	33.5069	0.184169
50	newly 3 weeks spec	943.	1720.08	{161., 3580.}	135.094	0.426698	newly 3 weeks cumul	1533.	2588.01	{362., 4912.}	116.276	0.353674	newly 5 weeks spec	5024.	5823.01	{433., 14 581.}	87.8445	0.512854	newly 5 weeks cumul	8966.	11 900.9	{1254., 32 720.}	98.006	0.454707	newly 65 weeks cumul	98 082.	54 341.3	{15 079., 92 324.}	44.5961	0.377341
